# The differential effects of leukocyte-containing and pure platelet-rich plasma (PRP) on tendon stem/progenitor cells - implications of PRP application for the clinical treatment of tendon injuries

**DOI:** 10.1186/s13287-015-0172-4

**Published:** 2015-09-15

**Authors:** Yiqin Zhou, Jianying Zhang, Haishan Wu, MaCalus V. Hogan, James H-C. Wang

**Affiliations:** MechanoBiology Laboratory, Department of Orthopaedic Surgery, University of Pittsburgh School of Medicine, 210 Lothrop Street, BST, E1640, Pittsburgh, PA 15213 USA; Joint Surgery and Sports Medicine Department, Shanghai Changzheng Hospital, Second Military Medical University, 415 Fengyang Road, Huangpu, Shanghai, 200003 China

## Abstract

**Introduction:**

Platelet-rich plasma (PRP) is widely used to treat tendon injuries in clinics. These PRP preparations often contain white blood cells or leukocytes, and the precise cellular effects of leukocyte-rich PRP (L-PRP) on tendons are not well defined. Therefore, in this study, we determined the effects of L-PRP on tendon stem/progenitor cells (TSCs), which play a key role in tendon homeostasis and repair.

**Methods:**

TSCs isolated from the patellar tendons of rabbits were treated with L-PRP or P-PRP (pure PRP without leukocytes) *in vitro*, followed by measuring cell proliferation, stem cell marker expression, inflammatory gene expression, and anabolic and catabolic protein expression by using immunostaining, quantitative real-time polymerase chain reaction, Western blot, and enzyme-linked immunosorbent assay, respectively.

**Results:**

Cell proliferation was induced by both L-PRP and P-PRP in a dose-dependent manner with maximum proliferation at a 10 % PRP dose. Both PRP treatments also induced differentiation of TSCs into active tenocytes. Nevertheless, the two types of PRP largely differed in several effects exerted on TSCs. L-PRP induced predominantly catabolic and inflammatory changes in differentiated tenocytes; its treatment increased the expression of catabolic marker genes, matrix metalloproteinase-1 (*MMP-1*), *MMP-13*, interleukin-1beta (*IL-1β*), *IL-6 *and tumor necrosis factor-alpha (*TNF-α*), and their respective protein expression and prostaglandin E_2_ (*PGE*_2_) production. In contrast, P-PRP mainly induced anabolic changes; that is, P-PRP increased the gene expression of anabolic genes, alpha-smooth muscle actin (*α-SMA*), collagen types I and III.

**Conclusions:**

These findings indicate that, while both L-PRP and P-PRP appear to be “safe” in inducing TSC differentiation into active tenocytes, L-PRP may be detrimental to the healing of injured tendons because it induces catabolic and inflammatory effects on tendon cells and may prolong the effects in healing tendons. On the other hand, when P-PRP is used to treat acutely injured tendons, it may result in the formation of excessive scar tissue due to the strong potential of P-PRP to induce inordinate cellular anabolic effects.

## Introduction

Acute and chronic tendon injuries affect millions of people in both occupational and athletic settings each year. Healing of acute injuries results in the formation of scar tissue in tendons, which have inferior mechanical strength that makes them susceptible to re-injury [1]. On the other hand, the current treatment of chronic tendon injury (or tendinopathy) is largely palliative because of the incomplete understanding of the tendon disorder [2]. In recent years, a new treatment option involving the injection or implantation of platelet-rich plasma (PRP) has been used in orthopaedic surgery and sports medicine to treat tendon injuries [3–5].

PRP is the plasma fraction derived from a person’s own blood and contains high concentrations of platelets that house a sleuth of growth factors such as platelet-derived growth factor, transforming growth factor-beta, vascular endothelial growth factor, and hepatocyte growth factor, which are known to play a critical role in tissue healing [6, 7]. After a simple centrifugation process, the platelet-containing PRP is injected into the injured area during the treatment protocol. PRP has several distinct advantages: it is autologous and biocompatible, making it inherently safe; it contains high levels of growth factors that promote healing of injured tissues; and when injected *in vivo*, it forms a fibrin scaffold, which is conducive for cell migration and new matrix formation [2, 8]. Because of these advantages, PRP has been used widely to promote bone formation [9], skin rejuvenation [10], and colon anastomosis [11] among other things. PRP’s safety and ease of use have also promoted its use among professional athletes to treat soft tissue injuries without hospitalization [12–14], allowing a quicker return to sports activities. Particularly, PRP treatment has been successful in healing injured anterior cruciate ligament, which is known to lack spontaneous healing ability [15–17].

Despite its widespread use, the efficacy of PRP treatment on tendon injuries particularly in clinical trials has been controversial. This is mainly due to inconsistent results from human clinical trials, and a number of studies have reported that PRP can effectively treat tendon injuries [12, 18–22] whereas others have contradicted the positive outcomes of PRP and noted no improvement in pain or tendon function after PRP treatment [23–26]. These controversies are likely due to PRP-related factors such as variations in preparations and dosage, and patient-related factors such as age, gender, disease history, and treatment history of patients among others [27]. One critical component that affects PRP preparations is the presence or absence of white blood cells (WBCs) or leukocytes (neutrophils, monocytes, macrophages, and lymphocytes), which can be beneficial because they stimulate the immune response against infections [28, 29]; promote chemotaxis, proliferation, and differentiation of cells; and induce extracellular matrix production and angiogenesis [30]. Owing to these properties, PRP-containing leukocytes (L-PRP) are often used to treat traumatic injuries [31]. However, leukocytes also release inflammatory cytokines—e.g., interleukin-1beta (*IL-1β*) and tumor necrosis factor-alpha (*TNF-α*)—and reactive oxygen species, both of which can have detrimental effects on the treated tissues [32]. Therefore, it is necessary to define the precise effects of L-PRP on tendons so that its clinical applications can be justified or refuted.

In tendons, two cell types exist: tenocytes, which are the dominant cells, and the tendon stem/progenitor cells (TSCs), which form a small portion (<5 %) of the total tendon cells [33]. Similar to other adult stem cells, TSCs self-renew and can differentiate into tenocytes that are responsible for the maintenance of the tendons and repair once injured [34]. Because TSCs play such a critical role in tendon homeostasis and repair, we designed this study to determine the effects of L-PRP on TSCs. Specifically, we tested the hypothesis that L-PRP and P-PRP (pure PRP without leukocytes) exert differential effects on TSCs, which may lead to different outcomes of PRP treatment on tendon injuries.

## Methods

### Preparation of L-PRP and P-PRP

The protocol for the use of rabbits in this study was approved by the University of Pittsburgh Institutional Animal Care and Use Committee. In total, 12 adult New Zealand White rabbits (6–8 months old, 3.0–4.0 kg) were used in this study. L-PRP and P-PRP were prepared in a two-step centrifugation process in accordance with a previously described protocol [35]. Briefly, whole blood was centrifuged at 2300 *g* for 10 s per milliliter of blood. Then, the top two thirds of the supernatant were obtained for P-PRP preparation while the lower one third of the supernatant and buffy coat were collected for L-PRP preparation. Both supernatants were centrifuged separately for a second time at 2200 *g* for 2 min/ml and the resulting “pellets” were used as L-PRP and P-PRP, respectively. The supernatants known as platelet-poor plasma (PPP) were also collected. The platelet concentration in the two preparations was measured by using an automatic hematology analyzer (CELL-DYN Emerald; Abbott Laboratories, North Chicago, IL, USA) and adjusted to three times higher than the platelet level in whole blood with PPP. Prior to use in the experiments, both L-PRP and P-PRP were activated by adding 100 U/ml bovine thrombin (catalog #T4648; Sigma-Aldrich, St. Louis, MO, USA).

### Isolation of rabbit TSCs

TSCs from rabbits were isolated as described previously [36]. Briefly, rabbits that were used to collect blood as described above were euthanized, and the patellar tendons from two rabbits were harvested by cutting the tendons 5 mm from the distal and proximal ends each. The tendon sheath and surrounding paratenon were carefully stripped, and the core part of each tendon was isolated, weighed, and minced into fragments (1 mm × 1 mm × 1 mm) for cell culture. These tendon fragments were digested in a solution containing 3 mg/ml collagenase type 1 (Worthington Biochemical Corporation, Lakewood, NJ, USA) and 4 mg/ml dispase (StemCell Technologies Inc., Vancouver, BC, Canada) in phosphate-buffered saline (PBS) at 37 °C for 1 h. The final suspension was centrifuged at 1500 *g* for 15 min, and the resulting cell pellet was suspended in growth medium consisting of Dulbecco’s modified Eagle’s medium (DMEM), 20 % fetal bovine serum (FBS) (Atlanta Biologicals, Lawrenceville, GA, USA), and 1 % each of penicillin and streptomycin. The cell suspension was diluted to 10 cells/μl, and about 2 × 10^4^ cells were seeded into six-well plates and incubated at 37 °C with 5 % O_2_. After 2 weeks, the TSC colonies observed in the wells were detached with trypsin (0.25 %) and transferred to T25 flasks for further culture in growth medium. These TSCs at passage 2 were used in further experiments. Prior to use, the stemness of TSCs was verified by staining the cells for stem cell markers, including *Oct-4*, *SSEA-4*, and nucleostemin (*NS*), as described previously [34].

### *In vitro* culture of TSCs

A transwell system (Transwell, Corning Incorporated, Corning, NY, USA) was used to culture cells for all the experiments in this study. This system consists of transwell inserts containing a microporous membrane with 0.4-μm pore size that can be inserted into the well of cell culture plates such that there is free flow of culture medium between the upper and lower compartments. First, TSCs in culture medium (DMEM + 2 % FBS) were seeded in the lower compartment, the cell culture well. Activated L-PRP or P-PRP in DMEM + 2 % FBS was then added to the upper compartment of the experimental groups. The control group received DMEM + 2 % FBS. The culture medium was collected and changed every 3 days. The optimal concentration of L-PRP and P-PRP required for the best growth of TSCs was determined by cell proliferation assay.

### Tendon cell proliferation assay

TSCs were seeded in a 24-transwell system (Transwell, catalog no. 3413; Corning Incorporated) at a density of 1 × 10^4^ and cultured in growth medium containing L-PRP or P-PRP at various concentrations: 0 %, 2 %, 5 %, 10 %, 20 % (vol/vol). Cell growth was evaluated on day 3 by using the Cell Counting Kit-8 (CCK-8) (Sigma-Aldrich, catalog no. 96992). Fresh culture medium containing 10 % CCK-8 solution was added to cells and incubated at 37 °C for 2.5 h. Then, the absorbance was measured by using a microplate reader (Spectra MAX 190; Molecular Devices, Sunnyvale, CA, USA) at 450 nm. Each treatment had three replicates and their absorption was independently calculated as OD 450nm_experiment_ – OD 450nm_blank_. The average absorption of the three replicates represented cell proliferation in each treatment group. The PRP concentration, which induced the highest level of cell proliferation, was considered the optimal concentration and therefore was used in the following experiments.

### Cell morphology

TSCs were seeded in a 24-transwell system at a density of 1 × 10^4^ per well and incubated in growth medium (DMEM + 2 % FBS) alone (control group) or growth medium with 10 % L-PRP or 10 % P-PRP (experimental groups). The cell culture medium was changed every 3 days. After 14 days, cell morphology was first observed microscopically and images were obtained through a camera attached to the microscope.

### Immunostaining of tendon cells

TSCs cultured as above for 14 days were collected by trypsinization, fixed for 20 min in 4 % paraformaldehyde/PBS, and washed with 0.1 % Triton-X100/PBS for 5 min. Immunostaining for the stem cell marker *NS* was performed by first blocking the fixed cells with 3 % goat serum for 1 h and then incubating in goat anti-rabbit *NS* antibody (1:500; Neuromics, Edina, MN, USA; catalog no. GT15050) for 1 h at room temperature, followed by incubation for the same time with a Cy3-conjugated donkey anti-goat immunoglobulin G (IgG) secondary antibody (Millipore; catalog no. AP180S). Nuclei in the cells were counterstained with Hoechst 33342 (Sigma-Aldrich; catalog no. 33270). Immunostaining for tenocyte-specific proteins, *α-SMA*, and collagen types I and III was performed by blocking the fixed cells in 2 % mouse serum. Cells were then incubated with mouse monoclonal anti-alpha-smooth muscle actin (anti-*α-SMA*) antibody (1:500; Abcam, catalog no. ab7817, Cambridge, UK), anti-collagen type I antibody (1:200; Abcam, catalog no. ab6308), or anti-collagen type III antibody (1:200; catalog no. NBP1-05119; Novus, Littleton, CO, USA) overnight. After washing in PBS three times for 5 min each, the cells were further incubated with Cy3-conjugated goat anti-mouse IgG secondary antibody (1:500; Millipore; catalog no. AP124X4-K) for 1.5 h. After a final wash in PBS, the stained cells were examined through an inverted fluorescence microscope (Nikon eclipse, TE2000-U; Nikon Inc., Melville, NY, USA) and images were obtained with a charge-coupled device camera using the SPOT imaging software (Diagnostic Instruments, Inc., Sterling Heights, MI, USA).

### Gene Expression Analysis using real-time qRT-PCR

To determine the effects of L-PRP and P-PRP on the gene expression in tendon cells, we used real-time quantitative reverse transcription-polymerase chain reaction (qRT-PCR) to analyze the following genes as previously described [37, 38]: stem cell-related gene (*Oct-4*), tenocyte-related genes (*α-SMA* and collagen type I and collagen type III), non-tenocyte related genes (*Sox-9, Runx-2* and *PPAR-γ*), catabolic genes (*MMP-1* and *MMP-13*), and inflammatory marker genes (*IL-1β, IL-6*, and *TNF-α*). First, TSCs were seeded in a six-transwell system at a density of 2 × 10^5^ and cultured in the presence of 10 % L-PRP or 10 % P-PRP. Cells from each treatment were harvested on day 14 to isolate total RNA by using the RNeasy Mini Kit with an on-column DNase I digest (catalog no. 74104; Qiagen, Valencia, CA, USA). First-strand cDNA was then synthesized from 1 μg of total RNA by using Super Script II (Invitrogen, Grand Island, NY, USA) in the following conditions: 65 °C for 5 min, 4 °C for 1 min, 42 °C for 50 min, and finally 72 °C for 15 min. Then, qRT-PCR was carried out in a 25-μl reaction volume containing 2 μl of cDNA (100 ng RNA) as template and gene-specific primers by using the Qiagen QuantiTect SYBR Green PCR Kit (Qiagen; catalog no. 204143). All reactions were performed on a Chromo 4 Detector (MJ Research, Waltham, MA, USA) by incubating at 94 °C for 5 min, followed by 40 cycles of a three temperature program consisting of 1 min at 94 °C, 40 sec at 57 °C, and 40 sec at 72 °C, followed by a melt-curve analysis. All primers were synthesized by Invitrogen, and *GAPDH* (glyceraldehyde 3-phosphate dehydrogenase) was used as an internal control. The sequences of primers used in the reactions are listed in Table 1 and are based on previous publications [39–45]. Each reaction had at least three replicates, and the relative expression of each target gene was calculated by using the formula 2^−ΔΔCT^, where CT is the mean cycle threshold (n = 3) of each cDNA amplified and ΔΔCT = (CT_target_/CT_GAPDH_) _experiment_ – (CT_target_/CT_GAPDH_) _control_ [46].

**Table 1:** Primers used in quantitative reverse transcription-polymerase chain reaction for gene expression analysis

Gene	Primer Sequence		Reference
*Oct-4*	For	5′-CGA GTG AGA GGC AAC TTG G-3′	[42]
	Rev	5′-CGG TTA CAG AAC CAC ACA CG-3′	
*Sox-9*	For	5′-TGA ATC TCC TGG ACC CCT TC-3′	[44]
	Rev	5′-CCG TTC TTC ACC GAC TTC CT-3′	
*Runx-2*	For	5′-TGA TGA CAC TGC CAC CTG TG-3′	[44]
	Rev	5′-ACT CTG GCT TTG GGA AGA GC-3′	
*PPAR-γ*	For	5′-TGC AGG AGC AGA GCA AAG AAG-3′	[45]
	Rev	5′-GAG GCC AGC ATG GTG TAG ATG-3′	
*MMP-1*	For	5′-ATA CCT GGA AAA CTA CTA CAA TCT G-3′	[39]
	Rev	5′-TCT TCA GGG TTT CAG CAT CT-3′	
*MMP-13*	For	5′-TGC CCC TCC TCA ACA GTA AC-3′	[39]
	Rev	5′-GAG CCC GCT GCA TTC TTC TT-3′	
*IL-1β*	For	5′-GTC GTT GTG GCT CTG GAG AA-3′	[41]
	Rev	5′-TGC CAG ACA ACA CCA AGG AT-3′	
*IL-6*	For	5′-CTG GTG GTG GCT ACC GCT TT-3′	[39]
	Rev	5′-ATG GTC TCC AGG ATG CTC CG-3′	
*TNF-α*	For	5′-CAG CCT CTT CTC TTT CCT GCT-3′	[39]
	Rev	5′-CCG ATC ACC CTG AAG TGC-3′	
*GAPDH*	For	5′-AAG GCC ATC ACC ATC TTC CA-3′	[39]
	Rev	5′-GGA TGC GTT GCT GAC AAT CT-3′	

### Western blot analysis

TSCs were cultured in the presence of 10 % L-PRP or 10 % P-PRP as above. The cell culture medium was changed every 3 days. After 14 days, they were harvested by using trypsin and centrifuged to obtain cells from each group. Total proteins were then isolated by lysing cells by using a mammalian protein extraction reagent (M-PER) (Thermo, Waltham, MA, USA, catalog no. 78505) containing 1.5 % protease inhibitors (Sigma-Aldrich) followed by centrifugation at 14,000 *g* for 10 min. The resulting supernatant was stored at 4 °C. Protein concentration in the supernatant was determined by using the BCA Protein Assay Kit (Thermo, catalog no. 23225). Equal amounts of total protein from each group were then separated on 12 % SDS-PAGE gels (Thermo, catalog no. 25222) at a constant 100 V for 60 min. For Western blot analysis, proteins were transferred to a nitrocellulose membrane by using a semi-dry transfer module (Bio-Rad Laboratories, Hercules, CA, USA) at 200 mA for 90 min. The membrane was blocked in 5 % dry milk/TBS-Tween 20 for 1 h at room temperature and then incubated overnight at room temperature with a mouse monoclonal anti-*α-SMA* antibody (Abcam, catalog no. ab7817) at a dilution of 1:1000, anti-collagen type I antibody (Abcam, catalog no. ab6308) at a dilution of 1:250, or anti-collagen type III antibody (Novus, catalog no. NBP1-05119) at a dilution of 1:250. The membranes were washed in PBS/Tween-20 three times for 10 min each and further incubated with peroxidase-conjugated goat anti-mouse antibody (Santa Cruz Biotechnology, Dallas, TX, USA, catalog no. sc-2005) at a dilution of 1:2500 in 1 % dry milk/PBS for 1 h at room temperature. Finally, the protein bands were detected by using an ECL (enhanced chemiluminescence) detection kit (Amersham Biosciences, Piscataway, NJ, USA), followed by exposing the membrane to x-ray films for visualization. Mouse anti-human *GAPDH* (Chemicon, Temecula, CA, USA) was used as an internal control to verify the loading of equal amounts of proteins in each well. After the protein bands on Western blots were obtained, semi-quantification was performed by using ImageJ (National Institutes of Health, Bethesda, MD, USA).

### Quantifying total collagen, *MMP-1*, and *MMP-13* production

TSCs were seeded in a six-transwell system at a density of 2 × 10^5^ per well and incubated in DMEM + 2 % FBS (control group) or DMEM + 2 % FBS + 10 % L-PRP or 10 % P-PRP (experimental groups). Each group had at least three replicates. The cell culture medium was changed every 3 days. After 7 days, the supernatant was collected from each well, and cells were detached by trypsinization and counted by using an auto cellometer (Cellometer Auto T4; Nexcelom Bioscience LLC, Lawrence, MA, USA). Total collagen production in each well was measured (90 % confluent) by using the Sircol collagen assay kit (Biodye Science, Biocolor Ltd., Carrickfergus, Northern Ireland, UK). In a separate culture, *MMP-1* and *MMP-13* levels in the supernatant were also measured after 4 days by using enzyme-linked immunosorbent assay (ELISA) assay kits in accordance with the instructions of the manufacturer (*MMP-1* and *MMP-13*; Biotang Inc., Boston, MA, USA).

### Measuring *IL-1β*, *IL-6*, and *TNF-α*

First, TSCs were cultured as above either in growth medium alone (control) or in growth medium containing 10 % L-PRP or 10 % P-PRP with fresh medium replenished every third day in culture. At least three replicates were maintained for each group. After 4 days, cells were collected by using trypsin and centrifuged; the pellet was used to estimate the cell count by using an auto cellometer (Cellometer Auto T4; Nexcelom Bioscience LLC), and the supernatant was used to measure *IL-1β*, *IL-6*, and *TNF-α* levels by using specific ELISA kits in accordance with the instructions of the manufacturer: *IL-1β* - RayBio Rabbit* IL-1beta* ELISA Kit (RayBiotech, Norcross, GA, USA); *IL-6* - Rabbit Interleukin 6, *IL-6* ELISA Kit (Cosmo Bio USA, Carlsbad, CA, USA); and *TNF-α* - Rabbit Tumor Necrosis Factor alpha ELISA Kit (MyBioSource, San Diego, CA, USA).

### Measuring *PGE*_2_ production

TSCs were cultured as above in growth medium containing 10 % L-PRP or 10 % P-PRP for 4 days with the medium changed every third day in culture. Three replicates were maintained for each group. The cell count in the culture flasks was measured by using an auto cellometer (Cellometer Auto T4; Nexcelom Bioscience LLC) after trypsinization. *PGE*_2_ level in the supernatant was measured by using the *PGE*_2_ ELISA assay kit in accordance with the instructions of the manufacturer (Cayman Chemical, Ann Arbor, MI, USA).

### Statistical analysis

Results are presented as the mean ± standard deviation. Statistical significance of the results from cell proliferation experiments was analyzed by performing one-way analysis of variance followed by Fisher’s protected least significant difference for multiple comparisons. For statistical analysis of other results, two tailed, paired, or unpaired Student’s *t* test was performed wherever applicable. Differences between two groups (control, L-PRP, or P-PRP) were considered significant when *P* values were below 0.05.

## Results

### Characterization of L-PRP and P-PRP preparations

Platelet concentrations in the L-PRP and P-PRP preparations were similar with 8.94–9.81 × 10^5^/μl and 8.87–9.75 × 10^5^/μl, respectively. These platelet concentrations are about three times higher than the average platelet level in rabbit whole blood (3.12 × 10^5^/μl). As expected, the two preparations differed largely in the amounts of WBCs present in them; WBC concentration in L-PRP preparations ranged between 7.1 and 10.5 × 10^3^/μl, which is higher than the mean WBC concentration in whole rabbit blood 4.87 × 10^3^/μl. However, the WBC concentration in P-PRP was negligible (<100/μl).

### Tendon cell proliferation rate is PRP dose-dependent

**Fig. 1:**
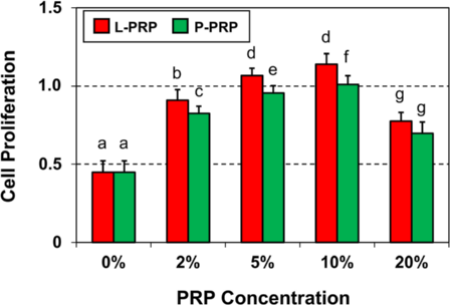
Proliferation of tendon stem/progenitor cells cultured in various concentrations of L-PRP or P-PRP. Cell proliferation was measured on day 3 in culture. Cells proliferated in a dose-dependent manner with 10 % L-PRP and 10 % P-PRP inducing the maximum effects. All data are mean ± standard deviation of three values. One-way analysis of variance followed by least significant difference was used for statistical analysis of the data from each group (L-PRP or P-PRP). For comparing differences between L-PRP and P-PRP, a *t* test was used to determine statistical significance. Note that different letters above bars indicate significant differences (*P* < 0.05). *L-PRP* leukocyte-platelet-rich plasma, *P-PRP* pure-platelet-rich plasma, *PRP* platelet-rich plasma

When TSCs were cultured in the presence of L-PRP or P-PRP, they differentiated (see data below), which are hitherto referred to as tendon cells or tenocytes. In the presence of L-PRP or P-PRP, tendon cell proliferation rate increased in a PRP dose-dependent manner (Fig. 1). Tendon cell proliferation increased by 46 % in the presence of 2 % P-PRP and more than 50 % at 5 % and 10 % P-PRP levels each. However, increasing P-PRP dose to 20 % decreased cell proliferation rate 31 % lower than 10 % P-PRP but still 36 % higher than the control cells without P-PRP (0 %). Similar results were observed in the presence of L-PRP with a maximum cell proliferation rate of 61 % observed at 10 % L-PRP level. In addition, 20 % L-PRP had the lowest cell proliferation rate (42 %) (Fig. 1). At each concentration, L-PRP treatment induced significantly higher cell proliferation than P-PRP. Since maximum proliferation rate was induced by 10 % L-PRP or 10 % P-PRP, this dose was used for further analyses.

### PRP specifically induces tenocyte differentiation of TSCs *in vitro*

**Fig. 2:**
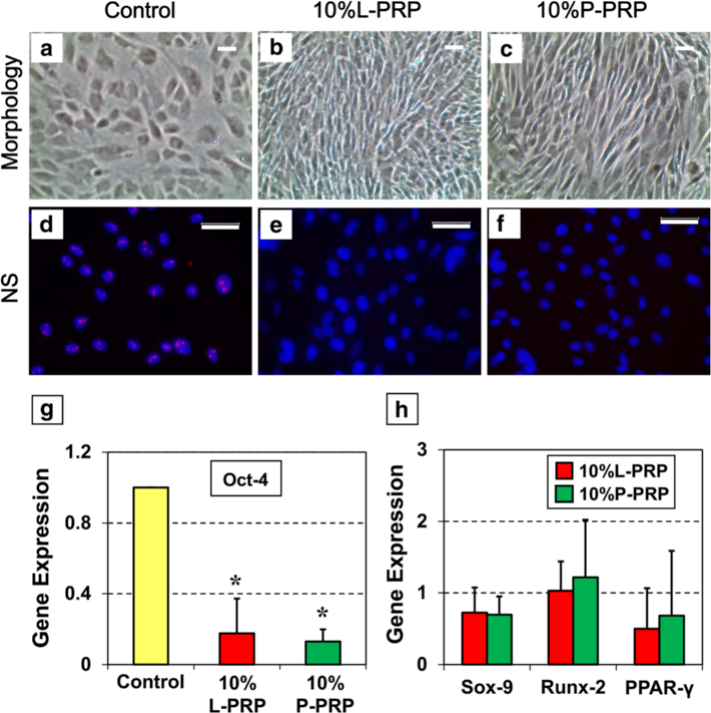
L-PRP and P-PRP induce TSC differentiation into tenocytes. Morphology of TSCs after 14 days in culture (**a**-**c**). In the control (**a**), cells were cobblestone-shaped, a typical feature of TSCs. But PRP treatment changed cell morphology into more elongated tenocyte-like cells and increased the cell numbers (**b**, **c**). Immunostaining for the stem cell marker nucleostemin (*NS*) (**d**). *NS* staining was positive in the control (**d** - pink dots) but negative in the PRP-treated cells (**e**, **f**). Quantitative reverse transcription-polymerase chain reaction analysis (**g**, **h**). Expression of the stem cell marker gene, *Oct-4*, was reduced in PRP-treated cells (**g**); however, PRP-induced changes on the expression of non-tenocyte genes, *Sox-9*, *Runx-2*, and *PPAR-γ*, were minimal (**h**). Gene expression levels were normalized with respect to the expression *GAPDH* (glyceraldehyde 3-phosphate dehydrogenase). Asterisks indicate significant differences (*P* < 0.05) when compared with the control. Statistical analyses were performed by using *t* test with a sample size of at least three in each group. All analyses were performed on cells in culture for 14 days. Bars = 100 μm (**a**-**f**). *L-PRP* leukocyte-platelet-rich plasma, *P-PRP* pure-platelet-rich plasma, *PRP* platelet-rich plasma, *TSC* tendon stem/progenitor cell

Microscopic observations of tendon cell morphology in the controls revealed the presence of cobblestone-shaped cells, which is typical for TSCs (Fig. 2a). However, treatment of TSCs with 10 % L-PRP or 10 % P-PRP not only increased the number of cells in culture but also specifically increased the numbers of elongated cells, which is the typical shape of tenocytes, the dominant resident cells in tendons (Fig. 2b, c). In addition, the number of cells was evidently higher after treatment with L-PRP than with P-PRP (Fig. 2b, c). Furthermore, immunostaining for the stem cell marker, *NS* (pink dots in Fig. 2d), showed a higher number of *NS*-positive cells in the control group versus the L-PRP- or P-PRP-treated groups (Fig. 2d-f). Besides, analysis of the *Oct-4* gene, a TSC marker by qRT-PCR, revealed a significant reduction in gene expression after PRP treatment when compared with the control (Fig. 2g), indicating that PRP decreases the stemness of TSCs *in vitro*. However, neither PRP preparations significantly increased or decreased the expression of non-tenocyte-specific genes (*Sox-9*, *Runx-2*, and *PPAR-γ*) (Fig. 2h) when compared with the control. These data indicate that both L-PRP and P-PRP preparations induce specific tenocyte differentiation of TSCs *in vitro.*

### Differentiated TSCs (tenocytes) are active

**Fig. 3:**
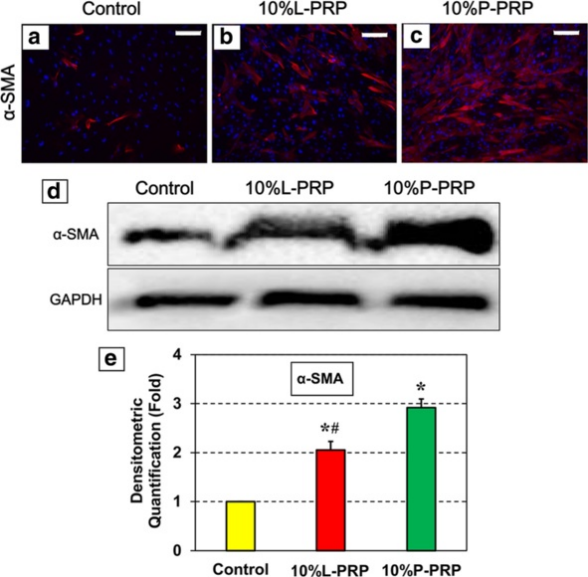
Tenocytes differentiated from L-PRP- or P-PRP-treated tendon stem/progenitor cells are active. Immunostaining for *α-SMA* (**a**-**c**), which is a marker of active tenocytes. PRP treatment increased the expression of *α-SMA* (pink/red stain) with higher staining after P-PRP treatment than with L-PRP. Nuclei are stained blue with Hoechst 33342. Western blot analysis (**d**). An intensely stained *α-SMA* protein band after PRP treatment validated increased *α-SMA* protein level albeit P-PRP induced more *α-SMA* production than L-PRP. Semi-quantification of the Western blots by ImageJ (**e**). At least three independent experiments were performed for each analysis. A *t* test was used to perform statistical analysis. Significant differences (*P* < 0.05) between each treatment and the control are indicated by asterisks. The pound sign indicates significant differences between L-PRP and P-PRP treatments. All analyses were performed after 14 days in culture. Bars = 200 μm. *α-SMA* alpha-smooth muscle actin, *L-PRP* leukocyte-platelet-rich plasma, *P-PRP* pure-platelet-rich plasma

We then determined whether the tenocytes newly formed by PRP-induced TSC differentiation were active in terms of collagen production. We first investigated the expression of the active tenocyte marker protein, *α-SMA*, in TSCs cultured in the presence of 10 % L-PRP or 10 % P-PRP. Immunostaining showed that PRP treatment increased the amounts of *α-SMA* when compared with control (Fig. 3a-c) with maximum staining observed in cells treated with 10 % P-PRP (Fig. 3c). Western blot analysis also validated these results, revealing that treatment with 10 % P-PRP induced the maximum levels of *α-SMA* in cells while the effect of 10 % L-PRP was not as effective as P-PRP (Fig. 3d). Quantification of the protein bands on Western blots also confirmed these observations (Fig. 3e).

**Fig. 4:**
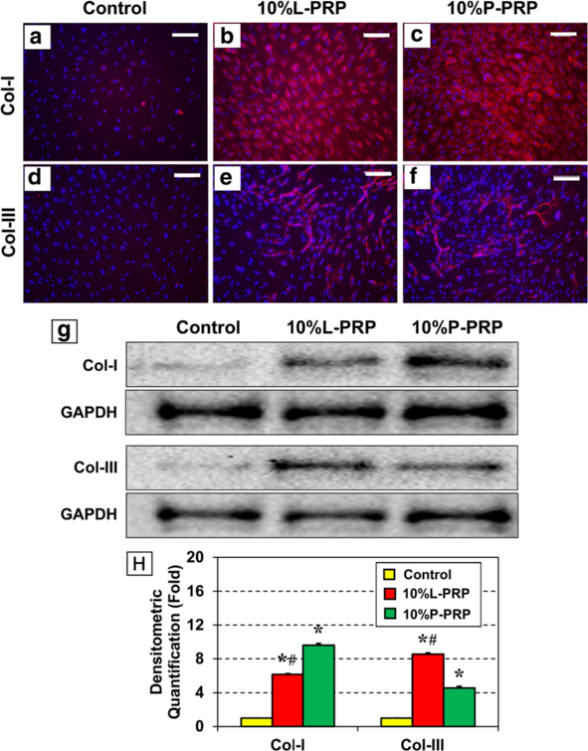
Active tenocytes differentiated from TSCs after L-PRP or P-PRP treatment express collagen types I and III. Immunostaining for collagen types I and III (**a**-**f**). Both PRP treatments increased the expression of collagen types I and III (pink/red stain), although cells treated with P-PRP stained more intensely for collagen type I and those treated with L-PRP stained more robustly for collagen type III. Nuclei are stained blue with Hoechst 33342. Western blot analysis (**g**) on total proteins extracted from cells cultured with L-PRP or P-PRP. Collagen I protein band was robust in the P-PRP-treated group; collagen III protein band was more abundant in the L-PRP-treated cells. Semi-quantification of the Western blots by ImageJ (**h**). Each data point represents at least three independent experiments. Statistical analyses were performed by using *t* test. Significant differences (*P* < 0.05) between each treatment and the control are indicated by asterisks. The pound sign indicates significant differences (*P* < 0.05) between L-PRP and P-PRP treatments. Analyses were performed after 14 days in culture. Bars = 200 μm. *Col* collagen, *GAPDH* glyceraldehyde 3-phosphate dehydrogenase, *L-PRP* leukocyte-platelet-rich plasma, *P-PRP* pure-platelet-rich plasma, *PRP* platelet-rich plasma, *TSC* tendon stem/progenitor cell

Furthermore, immunostaining of the tenocyte-related proteins, collagen types I and III, with specific antibodies displayed robust staining for both collagen types in the PRP-treated cells when compared with the control (Fig. 4a-f). In addition, staining for collagen type I was more intense than for collagen type III. These results were further confirmed by Western blot analysis where staining intensity of collagen type I after 10 % P-PRP treatment was much higher than treatment with 10 % L-PRP (Fig. 4g). However, treatment with 10 % L-PRP induced higher levels of collagen type III expression than 10 % P-PRP (Fig. 4g). These results were also corroborated by semi-quantification of the Western blots (Fig. 4h).

**Fig. 5:**
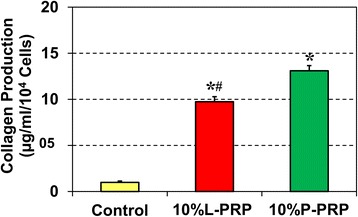
Tenocytes differentiated from L-PRP- or P-PRP-treated tendon stem/progenitor cells release abundant collagen into the culture media after 7 days. Quantification of total collagen was done by using the Sircol assay. As shown, both PRP preparations significantly stimulated the production of total collagen in these cells. The collagen production was normalized to the respective cell number in each group. The results are expressed as the mean ± standard deviation of three independent values in each group. A *t* test was used for statistical analysis. Asterisks indicate significant differences (*P* < 0.05) when compared with the control, and pound represents significant difference (*P* < 0.05) between L-PRP and P-PRP treatment. *L-PRP* leukocyte-platelet-rich plasma, *P-PRP* pure-platelet-rich plasma, *PRP* platelet-rich plasma

Furthermore, quantification of total collagen production in all three groups using the Sircol assay displayed significantly higher amounts of total collagen in the PRP-treated cells in comparison with the controls (Fig. 5). However, 10 % P-PRP (12-fold) induced greater amounts of total collagen production than 10 % L-PRP (nine-fold).

### L-PRP induces more extensive catabolic responses in differentiated tenocytes

**Fig. 6:**
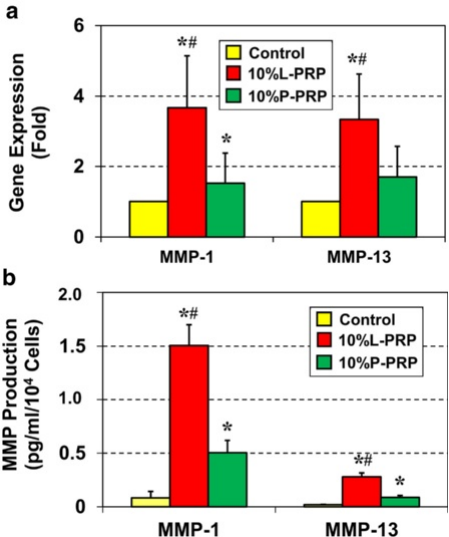
L-PRP induces greater catabolic responses than P-PRP. Gene expression analysis of the catabolic markers, *MMP-1* and *MMP-13*, by quantitative reverse transcription-polymerase chain reaction (**a**). Unlike P-PRP, L-PRP significantly induced the expression of both catabolic genes. The control group was used as the reference. Gene expression levels were normalized to the expression of *GAPDH* (glyceraldehyde 3-phosphate dehydrogenase). Data are represented as mean ± SD of three independent values. *MMP* production was also measured by using enzyme-linked immunosorbent assay (**b**). The production was normalized to respective cell number in each group. Asterisks represent significant differences when compared with the respective control, and the pound symbols indicate significant differences between L-PRP and P-PRP (*P* < 0.05). Note that the data for each group were calculated from three independent values. Data are expressed as the mean ± SD. A *t* test was used for statistical analysis. *L-PRP* leukocyte-platelet-rich plasma, *MMP* matrix metalloproteinase, *P-PRP* pure-platelet-rich plasma, *SD* standard deviation

We further investigated the effects of L-PRP and P-PRP on the expression of catabolic genes in the newly differentiated tenocytes. Treatment with 10 % L-PRP significantly upregulated the catabolic genes* MMP-1* and *MMP-13* compared with the untreated control, which was used as the reference (Fig. 6a). *MMP-1* registered a 4.0-fold increase when compared with the control, whereas *MMP-13* increased by approximately 2.4-fold. In addition, analysis of *MMP* production by ELISA was also in alignment with the gene expression results and revealed that 10 % L-PRP induced *MMP-1* and *MMP-13* levels approximately 15-fold higher than the control (Fig. 6b). Although 10 % P-PRP also upregulated the *MMPs* (*MMP-1*, 5-fold; *MMP-13*, 3.5-fold) when compared with the control, the increase was significantly less than that induced by 10 % L-PRP.

### L-PRP induces higher levels of inflammatory responses in differentiated tenocytes

**Fig. 7:**
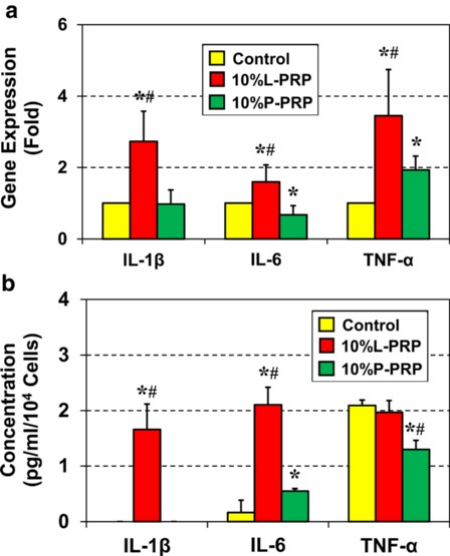
L-PRP produces greater inflammatory responses than P-PRP. Quantification of inflammatory marker gene expression was performed by using quantitative reverse transcription-polymerase chain reaction (**a**). Gene expression of *IL-1β*, *IL-6*, and *TNF-α* were upregulated by L-PRP. But P-PRP upregulated only *TNF-α*, downregulated *IL-6*, and did not have a significant effect on *IL-1β *gene expression (**a**). The control group was used as the reference (1-fold). Data are represented as mean ± SD of three independent values. Additionally, the levels of *IL-1β*, *IL-6*, and *TNF-α* were measured by using enzyme-linked immunosorbent assay (**b**). The concentration of each cytokine in each group was normalized to its cell number. L-PRP significantly increased *IL-1β* and *IL-6* protein levels but did not have a significant effect on *TNF-α* production. P-PRP did not affect *IL-1β*, increased *IL-6*, and decreased *TNF-α* protein levels in tenocytes differentiated from tendon stem/progenitor cells. Asterisks indicate significant differences between each PRP treatment and the respective control group (*P* < 0.05). Pound symbols represent significant differences between L-PRP- and P-PRP-treated groups (*P* < 0.05). Note that, for each group, three independent values were measured from three experiments, and the results are expressed as the mean ± SD. A *t* test was used for statistical analysis. *IL* interleukin, *L-PRP* leukocyte-platelet-rich plasma, *P-PRP* pure-platelet-rich plasma, *SD* standard deviation, *TNF-α* tumor necrosis factor-alpha

To investigate the effects of L-PRP and P-PRP on the inflammatory responses in the newly differentiated tenocytes, we first examined the expression levels of the inflammatory genes, *IL-1β*, *IL-6*, and *TNF-α*, by qRT-PCR. The results showed a significant increase in the expression of all three genes after treatment with 10 % L-PRP: *IL-1β* expression increased by 1.7-fold,* IL-6* by 0.6-fold, and *TNF-α* by approximately 2.4-fold (Fig. 7a). In contrast, P-PRP did not have any influence on the expression of *IL-1β*, decreased* IL-6* expression by 0.32-fold, and increased the expression of only *TNF-α *(0.93-fold) when compared with the control used as the reference (Fig. 7a). Moreover, the production level of the inflammatory cytokine *IL-1β* in the cell supernatants was consistent with the above gene expression data (Fig. 7b). Specifically, in the control culture,* IL-1β* was below detection level (45 pg/ml); however, treatment with 10 % L-PRP induced *IL-1β* levels, whereas 10 % P-PRP had no effect on *IL-1β*. *IL-6* levels increased 12-fold in the presence of 10 % L-PRP (Fig. 7b). The remaining protein expression data were not consistent with their gene expression results; P-PRP increased *IL-6* protein levels 2-fold and decreased *TNF-α* 0.6-fold; L-PRP did not change the protein level of *TNF-α* when compared with the control (Fig. 7b). Although the exact reasons for these results are not clear, they could be due to a high baseline level of *TNF-α* in the culture media.

**Fig. 8:**
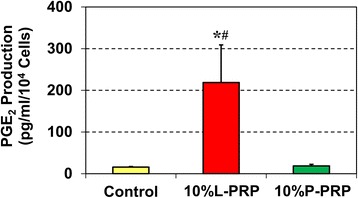
L-PRP enhances *PGE*_2_ production. Enzyme-linked immunosorbent assay results of *PGE*_2_ production by tenocytes after 4 days in culture. L-PRP induced higher production of *PGE*_2_ when compared with P-PRP. The *PGE*_2_ measurements in the three groups (control, L-PRP, and P-PRP) were normalized to respective cell numbers. Asterisk indicates comparison between each treatment and the respective control (*P* < 0.05). Pound sign indicates comparison between L-PRP and P-PRP (*P* < 0.05). Note that, for each group, three independent values were obtained, and the results are expressed as mean ± standard deviation. A *t* test was used for statistical analysis. *L-PRP* leukocyte-platelet-rich plasma, *PGE*_*2*_ prostaglandin E_2_, *P-PRP* pure-platelet-rich plasma

Furthermore, we investigated the production of *PGE*_2_ by tenocytes treated with 10 % L-PRP or 10 % P-PRP by using ELISA kits. The increase in *PGE*_2_ levels by L-PRP (13-fold) was apparent, but P-PRP treatment did not affect *PGE*_2_ levels in the newly differentiated tenocytes (Fig. 8).

## Discussion

PRP treatment is widely used to treat tendon injuries in clinics, although the efficacy of its treatment is a hotly debated topic. The use of PRP is supported by a number of human clinical trials as well as basic science studies on animal models and cell cultures [3, 4, 12, 22, 47]. Whereas basic science studies have generally confirmed that PRP promotes the healing of tendon injuries by enhancing tendon cell proliferation and anabolic activities, clinical studies have reported variable results in PRP treatment outcomes. There are many reasons for the conflicting reports [27]. In this study, we examined an important factor that may contribute to this inconsistency, which is the variation in PRP formulations that is due to differences in the preparation protocols with some resulting in L-PRP containing variable concentrations of leukocytes and some resulting in P-PRP without leukocytes.

Therefore, in this study, we investigated whether the presence of leukocytes in PRP affects the proliferation and differentiation of TSCs isolated from young adult rabbits. Our findings demonstrated that both L-PRP and P-PRP preparations induced TSC differentiation into active tenocytes, which were proliferating in culture in a PRP-dose-dependent manner (Figs. 1–5). Thus, neither L-PRP nor P-PRP appears to pose safety concerns, in terms of producing non-tendinous tissues in the treated tendons, for their use in clinics to treat tendon injuries. However, both PRP preparations differed in the following aspects: L-PRP treatment increased the expression of catabolic genes and proteins (*MMP-1*, *MMP-13*, *IL-1β*, *IL-6*, and *TNF-α*) and the production of *PGE*_2_, an inflammatory mediator in tendon cells that, at high concentrations, impairs tendon cell proliferation and induces non-tenocyte differentiation [48]. In contrast, P-PRP specifically induced differentiation of TSCs into active tenocytes, marked by *α-SMA* expression, and stimulated cellular production of collagen types I and III; more importantly, P-PRP affected *PGE*_2_ production minimally. These findings indicate that L-PRP and P-PRP exert differential effects on TSCs.

Therefore, the type of PRP preparation (L-PRP vs. P-PRP) is likely a critical factor in assessing the efficacy of PRP treatment on tendon injuries in clinics because they produce differential effects on tendon cells, as demonstrated by this and other studies. In clinics, PRP is prepared by using commercially available PRP preparation kits. Although most kits yield high platelet concentrations (as expected), the level of leukocytes in the PRP preparations may vary, thus likely contributing to variable treatment efficacies. Indeed, using two commercial kits (GPS and ACP) to prepare PRP, a recent study showed that active forms of *MMP*-2, -3, and -9 were present in both preparations that can induce catabolic effects on treated tissues and, as a result, could impair tissue healing [49].

The results of this study showed that the anabolic effects of L-PRP differed from P-PRP because L-PRP induced higher expression of collagen type III than P-PRP but lower expression of collagen type I than P-PRP. Because collagen type I is the principal component in tendons and collagen type III is present only in small amounts in normal tendons but large amounts in healing tendons with scars, these results indicate that the use of L-PRP to treat injured tendons may lead to scar formation in healing tendons. Moreover, L-PRP induces extensive catabolic responses in differentiated TSCs (tenocytes), which may delay the repair of acutely damaged tendon matrix and new matrix formation, thus slowing the healing of injured tendons. Last, because L-PRP induces inflammatory responses in tenocytes differentiated from TSCs, its use to treat the already-inflamed tendinopathic tendons may only exacerbate the tendon disorder by prolonging the inflammatory phase, thus impairing the healing process and leading to increased pain in patients. Caution should therefore be exercised when using PRP. Based on the data from this study, we suggest the use of P-PRP to augment the repair of tendinopathic tendons because of its anabolic properties and low inflammatory effects. On the other hand, it is plausible that the strong anabolic effects of P-PRP may cause fibrosis/scar tissue formation in acutely injured tendons simply because tenocytes differentiated from TSCs after P-PRP treatment produces too much collagen in the wound areas. In this situation, L-PRP with a small number of leukocytes may be beneficial because these inflammatory cells can induce catabolic effects on the treated tendons to balance out the excessive anabolic effects of P-PRP. Note that use of L-PRP with high levels of leukocytes may also lead to scar formation because of its ability to induce higher collagen type III production. Therefore, we suggest that whether to use L-PRP or P-PRP depends on the type of tendon injury (acute vs. chronic) and treatment phase (early- or late-stage healing) in clinical settings.

Previous studies have not specifically investigated the differential effects of L-PRP and P-PRP on TSCs. However, in an earlier study, we investigated the effects of PRCR (platelet-rich clot releasate) on TSCs. PRCR is comparable to P-PRP in this study except that it was obtained by activating platelets with calcium chloride instead of thrombin used in this study. We found that PRCR also promoted TSC differentiation into active tenocytes and their proliferation rate and collagen production *in vitro* [37]. Using an *in vivo* animal model, we further showed that PRP, or specifically PRCR in fibrin gel, reduced tendon inflammation [37]. Other studies have reported the effects of PRP on cell types other than tendon cells. Using human dermal fibroblasts (HDFs), for example, one study showed that the treatment of HDFs with 5 % L-PRP significantly increased cell proliferation and the expression of collagen type I and *MMP*-1 proteins [10]. Similarly, L-PRP treatment of human chondrocytes from osteoarthritic cartilage induced catabolic mRNA, particularly *IL-1β* and *IL-6*, whereas P-PRP stimulated chondrocyte anabolism by increasing the expression of collagen type II and aggrecan transcripts in chondrocytes [50]. These findings are consistent with our study on TSCs. Conversely, tendon injuries treated with both PRP and TSCs together have been shown to promote tendon healing better than each component alone [5, 51].

In this study, we found that both L-PRP and P-PRP had a dose-dependent effect on the proliferation of tenocytes differentiated from TSCs and that the optimal effect was achieved at 10 % L/P-PRP concentration. This dose-dependent effect is likely due to the increasing concentration of platelets in L-PRP and P-PRP. Similar observations were made by others where 10 % PRP induced the maximum proliferation of mesenchymal stem cells and a further increase suppressed cell proliferation [52, 53]. More importantly, a recent evaluation of various leukocyte-reduced PRP (lrPRP) concentrations on equine superficial digital flexor tendon illustrated that the beneficial effects of lrPRP plateaued at a certain platelet concentration. A further increase resulted in a significant reduction in collagen types I and III proteins, indicating that to promote tendon healing, decreasing the levels of leukocytes in PRP may be more critical than increasing platelet concentration [54].

Although L-PRP promoted the expression of catabolic genes in tenocytes differentiated from TSCs, it increased cell proliferation. This result is consistent with some previous studies, which reported the positive effects of leukocytes on cell proliferation [55, 56]. However, the increase in cell proliferation rate alone cannot be used to evaluate the effects of L-PRP on cells, because L-PRP is known mostly for its catabolic effects on cells and tissues; for example, L-PRP induced the highest levels of* IL-1β* and *TNF-α* in equine ficialis tendon explants [32] and increased the levels of* IL-1β* and *IL-6* in human chondrocytes [50]. These results are consistent with findings of this study indicating that L-PRP induces more catabolic effects on tendon cells, which could lead to detrimental effects on injured tendons, such as impaired healing.

Similar to our findings, the differential effects of L-PRP and P-PRP were recently demonstrated on human chondrocytes *in vitro* where L-PRP induced catabolic effects and P-PRP promoted anabolic effects [50]. In both studies, *IL-1β* and *IL-6* were upregulated by L-PRP but the effects of L-PRP on *TNF-α* differed; it was reported that higher* TNF-α* mRNA levels were observed after treatment with P-PRP when compared to treatment with L-PRP [50]. In this study, we found an opposite trend with L-PRP inducing higher amounts of *TNF-α* mRNA and protein than P-PRP. It is yet to be determined whether L-PRP could also differently influence various cell types (human chondrocytes vs. rabbit TSCs). A striking difference we noticed between the two studies is that, in the previous study, the platelet concentration in L-PRP was three times higher than in P-PRP [50], which makes it difficult to determine whether the adverse effects of L-PRP was due to the presence of leukocytes or high platelet concentration. In our study, the platelet concentration in P-PRP and L-PRP was comparable, allowing a direct comparison between the two types of PRP preparations. Our finding is also supported by McCarrel et al. [32], who reported that *TNF-α* gene expression levels were the highest when equine superficialis explants were treated with L-PRP in comparison with standard PRP (equivalent to P-PRP in this study) or PRP with highly concentrated platelets. Here, platelet concentrations in L-PRP and standard PRP were similar, supporting the hypothesis that the adverse effects of leukocytes in PRP on cells may be more than the deleterious effects of high platelet concentrations on cells.

## Conclusions

This study has revealed that both L-PRP and P-PRP induce the differentiation of TSCs into active tenocytes and increase their proliferation. However, L-PRP induced catabolic and inflammatory responses in differentiated tenocytes, whereas P-PRP mostly augmented anabolic responses. Therefore, we suggest that, because of its catabolic and inflammatory action, L-PRP should not be used in the treatment of chronic tendon injuries when chronic inflammation and degeneration are involved. Application of L-PRP in such conditions could only worsen tendon inflammation and degeneration, thus delaying healing of such tendon injuries. On the other hand, P-PRP may not be used to treat acutely injured tendons in young adults, because of its potential to induce the formation of excessive scar tissue due to its potent anabolic action. Therefore, the choice of PRP for a treatment should be determined by whether a tendon injury is acute or chronic and the treatment phase; L-PRP could benefit early-phase healing because of its ability to fight off infections, whereas P-PRP could be used for late-stage healing because of its anabolic effects, enabling it to augment and accelerate tendon healing. On the other hand, the proportion of leukocytes in PRP should be adjusted on the basis of the tendon condition. These insights explain, in part, the variable outcomes of PRP treatments in clinical trials and will improve future PRP treatments for tendon injuries.

## Abbreviations

*α-SMA*: Alpha-smooth muscle actin
CCK-8: Cell Counting Kit-8
CT: Cycle threshold
DMEM: Dulbecco’s modified Eagle’s medium
ELISA: Enzyme-linked immunosorbent assay
FBS: Fetal bovine serum
HDF: Human dermal fibroblast
IgG: Immunoglobulin G
*IL*: Interleukin
L-PRP: Leukocyte-platelet-rich plasma
lrPRP: leukocyte-reduced platelet-rich plasma
*MMP*: Matrix metalloproteinase
*NS*: Nucleostemin
OD: Optical density
PBS: Phosphate-buffered saline
*PGE*_2_: Prostaglandin E_2_
PPP: Platelet-poor plasma
P-PRP: Pure-platelet-rich plasma
PRCR: Platelet-rich clot releasate
PRP: Platelet-rich plasma
qRT-PCR: Quantitative reverse transcription-polymerase chain reaction
*TNF-α*: Tumor necrosis factor-alpha
TSC: Tendon stem/progenitor cell
WBC: White blood cell
